# A sensitivity indicator screening and intelligent classification method for the diagnosis of T2D-CHD

**DOI:** 10.3389/fcvm.2024.1358066

**Published:** 2024-04-24

**Authors:** Jiarui Li, Changjiang Ying

**Affiliations:** ^1^ The First Clinical Medical College, Xuzhou Medical University, Xuzhou, Jiangsu, China; ^2^ Department of Endocrinology, The Affiliated Hospital of Xuzhou Medical University, Xuzhou, Jiangsu, China

**Keywords:** type 2 diabetes mellitus, coronary heart disease, neural network model, logistic regression model, early detection

## Abstract

**Background:**

The prevalence of Type 2 Diabetes Mellitus (T2D) and its significant role in increasing Coronary Heart Disease (CHD) risk highlights the urgent need for effective CHD screening within this population. Despite current advancements in T2D management, the complexity of cardiovascular complications persists. Our study aims to develop a comprehensive CHD screening model for T2D patients, employing multimodal data to improve early detection and management, addressing a critical gap in clinical practice.

**Methods:**

We analyzed data from 699 patients, including 471 with CHD (221 of these also had T2D) and a control group of 228 without CHD. Employing strict diagnostic criteria, we conducted significance testing and multivariate analysis to identify key indicators for T2D-CHD diagnosis. This led to the creation of a neural network model using 21 indicators and a logistic regression model based on an 8-indicator subset. External validation was performed with an independent dataset from an additional 212 patients to confirm the models’ generalizability.

**Results:**

The neural network model achieved an accuracy of 90.7%, recall of 90.78%, precision of 90.83%, and an F-1 score of 0.908. The logistic regression model demonstrated an accuracy of 90.13%, recall of 90.1%, precision of 90.22%, and an F-1 score of 0.9016. External validation reinforced the models’ reliability and effectiveness in broader clinical settings.

**Conclusion:**

Our AI-driven diagnostic models significantly enhance early CHD detection and management in T2D patients, offering a novel, efficient approach to addressing the complex interplay between these conditions. By leveraging advanced analytics and comprehensive patient data, we present a scalable solution for improving clinical outcomes in this high-risk population, potentially setting a new standard in personalized care and preventative medicine.

## 1 Introduction

Type 2 diabetes (T2D) is a significant global health issue, currently affecting over 573 million individuals worldwide. This number is projected to increase, highlighting the urgency for effective management strategies to mitigate associated complications, including coronary heart disease (CHD) (1, 2). T2D is a major risk factor for the development of CHD, primarily due to the chronic hyperglycemic state it induces. This state exacerbates oxidative stress and inflammation, leading to damage of the coronary endothelium and the subsequent development of CHD (3).

Patients with T2D are 2–4 times more likely to develop CHD than those without diabetes, and they often present with more severe manifestations of the disease (4). This increased risk underscores the importance of early and accurate screening for CHD in the diabetic population. Despite the advancements in managing T2D, patients continue to face significant risks of cardiovascular complications. The American College of Cardiology notes that diabetic patients with acute coronary syndrome (ACS) are at a higher risk of severe post-percutaneous coronary intervention (PCI) complications, such as target lesion revascularization, myocardial infarction, and cardiovascular death, despite aggressive glucose-lowering strategies (5).

Moreover, large-scale clinical trials like ACCORD, ADVANCE, and VADT have highlighted the complexity of managing cardiovascular risks in patients with T2D, revealing that while strict glycemic control can reduce microvascular complications, it may not necessarily translate to a reduced risk of macrovascular events, such as CHD (6). This paradox further emphasizes the need for a proactive approach in screening for CHD among patients with T2D.

The diagnosis of CHD largely depends on coronary angiograms, a vital tool for visualizing the heart's blood vessels. However, in patients with both CHD and T2D, CHD symptoms may be masked by the neuropathic effects of high blood glucose levels, complicating the clinical presentation (7). Our research focuses on developing a comprehensive screening model for CHD among individuals with T2D. This model aims to utilize a wide array of multimodal data, including detailed patient histories, clinical examinations, and laboratory tests, to facilitate early detection and intervention for CHD in this high-risk population. By enhancing early detection efforts, we aspire to significantly improve patient outcomes and navigate the complexities associated with the co-management of T2D and CHD, thereby addressing a critical gap in current clinical practice.

## 2 Methods

### 2.1 Retrospective study design and patient data analysis

#### 2.1.1 Patient data collection

This research is grounded in a retrospective analysis, leveraging a comprehensive patient dataset from the Department of Cardiovascular Medicine at Xuzhou Medical University Affiliated Hospital. The dataset spans records over the last four years, including individuals diagnosed with Coronary Heart Disease (CHD) and Type 2 Diabetes Mellitus (T2D), aiming to develop an intelligent diagnostic model. This model capitalizes on a diverse array of clinical and biochemical indicators for its analysis.

##### 2.1.1.1 Diagnostic criteria

CHD was confirmed via coronary angiography, indicating stenosis exceeding 50% in at least one major coronary artery or over 70% in its primary branches.

T2D diagnosis followed the American Diabetes Association criteria, characterized by fasting plasma glucose levels exceeding 7.0 mmol/L or plasma glucose concentrations above 11.0 mmol/L two hours post-oral glucose tolerance testing (OGTT) (4).

##### 2.1.1.2 Exclusion criteria

Patients with severe valvular heart disease or non-ischemic myocardial disease were excluded from the study.

#### 2.1.2 Clinical and biochemical data inclusion

The study categorized examination indicators into clinical symptoms, biochemical markers, and ECG indicators, encompassing variables such as age, gender, duration of diabetes, history of hypertension, lipid profiles, and various ECG abnormalities.

For the biochemical indicator and ECG feature data, since the numerical distribution intervals of multiple indicators vary greatly, the min-max strategy is used for normalization processing. If the original data is distributed in the interval min,max, transform it to the interval ${\text{min}}^{\prime },\;{\text{max}}^{\prime }$, the formula is as follows:

(1) $$v^{\prime }={\text{min}}^{\prime }+\frac{v-\min}{\max-\min}({\text{max}}^{\prime }-{\text{min}}^{\prime }).$$

For clinical status information, the collected indicator information can be structured and quantified to form a standardized numerical vector.

#### 2.1.3 Ethical considerations

The study adhered to stringent ethical guidelines, with approval from the Medical Ethics Committee of Xuzhou Medical University Affiliated Hospital (Ethical Approval Number: XYFY-2022-0217) and Xuzhou Central Hospital (Ethical Approval Number: XCH-20240202). All patient data were anonymized and analyzed with utmost confidentiality to protect privacy.

### 2.2 Sensitive indicator screening based on significance testing

#### 2.2.1 Univariate analysis for preliminary screening

The initial patient pool included 471 individuals with CHD, among which 221 were also diagnosed with T2D (T2D-CHD), and 250 had CHD exclusively (CHD-only). Additionally, 228 individuals with T2D but without CHD (T2D-only) were incorporated for comparative analysis. A thorough univariate examination of each clinical and biochemical indicator was conducted to narrow down the extensive list of potential biomarkers to a more focused subset.

#### 2.2.2 Sensitive indicator combination screening

In this step, we integrated indicators into a discriminant model based on their individual significance. This process was refined through iterative testing to identify an optimal set of indicators for disease diagnosis. To thoroughly evaluate the impact of these indicator combinations on diagnostic outcomes, we employed several statistical measures:

(1) Logistic Regression Coefficient (*B*): This metric quantifies the relationship between each independent variable and the outcome, with its sign indicating the direction of correlation. The coefficient is determined as follows:
  (2) $$B=\frac{\sum (x_{i}-\bar{X})*(y_{i}-\bar{Y})}{\sum (x_{i}*y_{i})}.$$
(2) Odds ratio (OR), a statistical measure of the strength of association between two variables. OR can be calculated as:
  (3) $$OR=e^{B},$$
(3) Wald, is a chi-square statistic used to assess the effect of the independent variables on the dependent variable in a multiple indicator regression model. Wald can be calculated as:
  (4) $$Wald={\left(\frac{B}{SE_{B}}\right)}^{2},$$
  where $SE_{B}$ is the standard error of *B*.
(4) *P*-value, a statistic used to evaluate the results of the Wald test. The smaller the *p*-value, the more significant the association between the independent and dependent variables. The *P*-value can be calculated as:
  (5) $$P=1-\phi \left(\sqrt{Wald}\right),$$

where ϕ(⋅) denotes the normal distribution function.

### 2.3 Intelligent diagnostic model development and initial verification

#### 2.3.1 Neural network classification model

**Figure 1:**
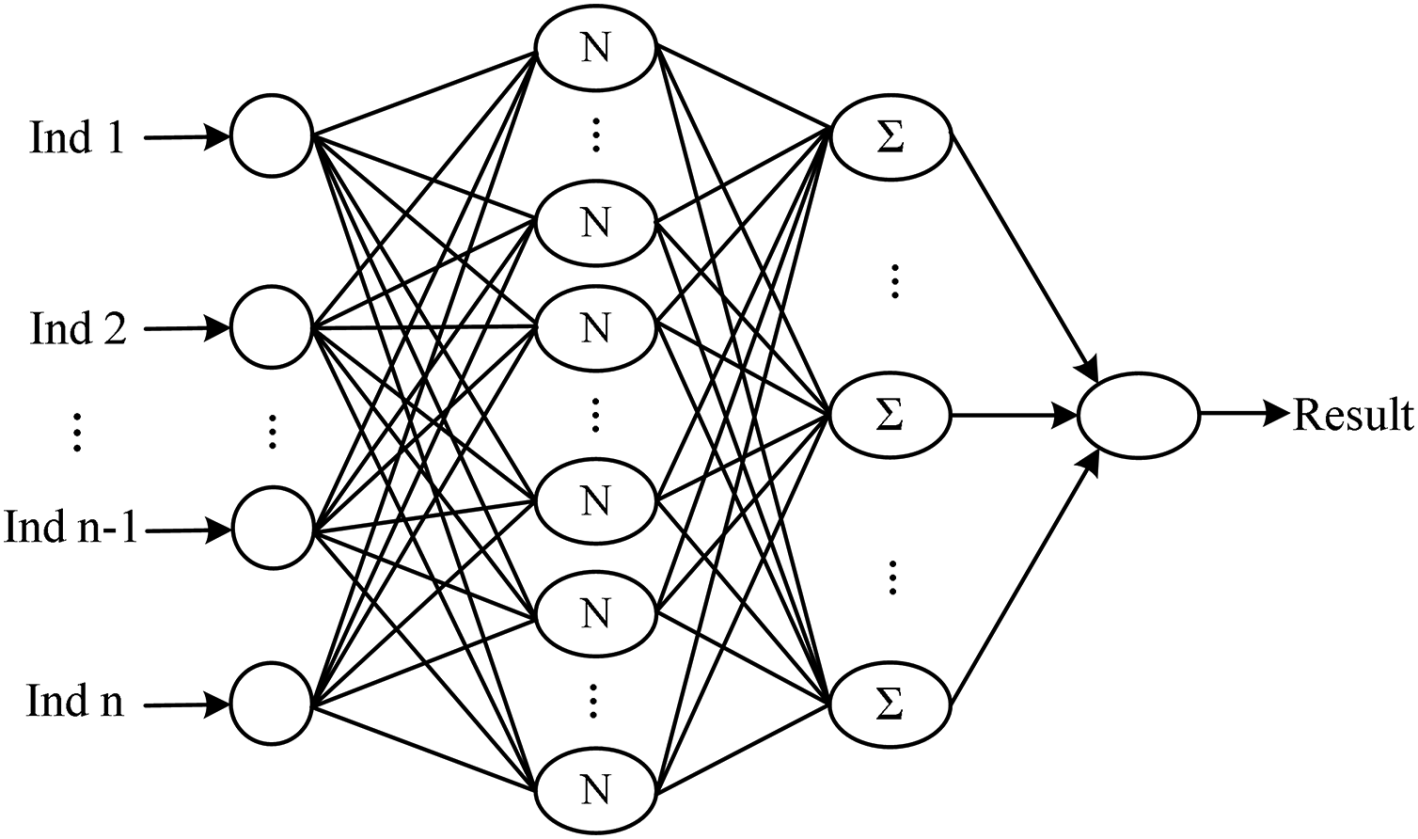
The architecture of the neural network classification diagnostic model. The model features a fully connected double-hidden-layer network structure, designed to enhance self-organizing learning and provide strong nonlinear mapping capabilities.

The neural network classification model, with a fully connected double-hidden-layer architecture, showcases strong self-organizing and nonlinear mapping capabilities, depicted in Figure 1. A dataset of 699 samples—221 T2D-CHD, 250 CHD-only, and 228 T2D-only—with 68.2% for training and the remainder for validation (Table 1), it uses 21 indicators to distinguish between patient groups effectively.

**Table 1:** Dataset partitioning.

	T2D-CHD	CHD-only	T2D-only	Sample size	Percentage
Training dataset	151	170	152	473	68.2%
Test dataset	70	80	76	226	31.8%
Total	221	250	228	699	

To optimize performance and mitigate overfitting, we employed a 25% Dropout regularization in the second hidden layer and selected cross-entropy loss, ideal for classification due to its precision in assessing output-label discrepancies (Table 2). The Mini-Batch Gradient Descent (MBGD) algorithm facilitated training, focusing on minimizing cross-entropy loss to improve accuracy (Table 3).

**Table 2:** Model parameter settings.

Input	Hidden layer			Output	
Node	Number of layers	Node	Activation function	Node	Activation function
17	2	12, 5	Tanh	1	Softmax

**Table 3:** Training parameter settings.

Loss function	Training algorithms	Batch size	Iterations	Learning rate	Regularization
Cross Entropy	MBGD	32	50	0.1	Dropout

Hyperparameter tuning followed a phased strategy, starting with broad adjustments to refine our search for the optimal hyperparameter set. This methodical tuning, alongside performance evaluation of each parameter set, guaranteed enhanced model performance on both training and validation datasets.

#### 2.3.2 Logistic regression classification model

The logistic regression classification model, designed to diagnose T2D-CHD, incorporates L2 regularization within a generalized linear regression framework to mitigate overfitting. This model estimates the probability of T2D-CHD occurrence by applying the logistic function to linear combinations of selected indicators, optimizing its coefficients and bias vector through supervised learning. The basic model is:

(6) y=L(W⋅X+B),

where, the coefficient matrix *W* and the bias vector *B* are the parameters to be solved, which are determined by supervised learning on the sample set.

Logistic regression uses the logistic function *L* to correspond W⋅X+B to the probability *p* of the occurrence of a hidden state, and then determines the value of the dependent variable based on the size of *p* and 1−p, that is, whether it is T2D-CHD.

#### 2.3.3 Evaluation metrics

The logistic regression model's evaluation will also comprehensively encompass Accuracy, Recall, Precision, and the F-1 Score. These added metrics, indispensable for evaluating the model's efficacy in accurately diagnosing T2D-CHD cases, will provide a more detailed view of the model's overall diagnostic performance.

(1) Accuracy represents the proportion of true results (both true positives and true negatives) among the total number of cases examined. It is the most intuitive performance measure and it gives an overall effectiveness of the model. Accuracy is calculated as follows:
  (7) $$\mathrm{Accuracy}=\frac{\mathrm{TP}+\mathrm{TN}}{\mathrm{TP}+\mathrm{TN}+\mathrm{FP}+\mathrm{FN}},$$
  Where TP is the number of true positives, TN is the number of true negatives, FP is the number of false positives, and FN is the number of false negatives.
(2) Recall, also known as sensitivity, measures the proportion of actual positive cases that were correctly identified. It is particularly important in medical diagnostics, where failing to identify a condition (false negative) can be more critical than incorrectly diagnosing it (false positive). Recall is defined as:
  (8) $$\mathrm{Recall}=\frac{\mathrm{TP}}{\mathrm{TP}+\mathrm{FN}},$$
(3) Precision assesses the proportion of positive identifications that were actually correct. It is crucial in situations where the cost of false positives is high. Precision is given by:
  (9) $$\mathrm{Precision}=\frac{\mathrm{TP}}{\mathrm{TP}+\mathrm{FP}},$$
(4) The F-1 Score is the harmonic mean of Precision and Recall, providing a balance between the two metrics. It is especially useful when the class distribution is imbalanced. The F-1 Score is calculated as:
  (10) $$F-1\;\;\mathrm{Score}=2\times \frac{\mathrm{Precision}\times \mathrm{Recall}}{\mathrm{Precision}+\mathrm{Recall}},$$

### 2.4 External validation

To enhance our statistical analysis and model validation, we expanded our dataset through collaboration with Xuzhou Central Hospital, incorporating data from an additional 73 patients with both T2D and CHD (T2D-CHD), 69 patients with CHD but without T2D (CHD-only), and 70 individuals with T2D but without CHD (T2D-only). This effort was aimed at increasing the dataset's diversity and improving the generalizability of our findings.

The external dataset underwent the same preprocessing and evaluation protocols as the initial dataset, ensuring a consistent and rigorous assessment.

### 2.5 Statistical analysis

Data analysis in our study was conducted using IBM SPSS Statistics 26, covering both descriptive and inferential statistics to address our research questions comprehensively. After preprocessing the data for quality, we employed descriptive statistics to summarize the data distribution. Inferential analysis followed, with one-way ANOVA and the Kruskal–Wallis H test applied to continuous variables to compare group means or medians based on normality checks. For categorical variables, the chi-square test evaluated the significance of distributions across groups. These methods collectively enabled the identification of significant disease indicators and their interactions, underpinning the development of our intelligent diagnostic model.

## 3 Result

### 3.1 Univariate analysis

Univariate analysis identified crucial indicators for diagnosing T2D in conjunction with CHD. Analysis results, as outlined in Table 4, indicated significant disparities in key indicators among three groups: those with T2D and CHD, those with CHD alone, and those with T2D only. Notable indicators included age, heart rate, HbA1c levels, fasting blood glucose, total cholesterol (TC), HDL-C, LDL-C, troponin I, creatinine, uric acid, albumin, γ-glutamyl transferase (GGT), total bilirubin, apolipoprotein A1, apolipoprotein B, T-wave changes, and ST-segment changes. The history of hypertension, aspartate aminotransferase (AST), and alanine aminotransferase (ALT) showed no significant variance, suggesting their limited value in differentiation.

**Table 4:** Results of univariate analysis.

Indicators	T2D-CHD (*n* = 151)	CHD-only (*n* = 170)	T2D-only (*n* = 152)	*P*-value
Age (year)	63 (45, 69)	68 (57.16, 73)	54.28 (47.82, 63)	<0.001
Heart rate	74 (65, 81)	71.3 (63, 77)	72.79 (63.95, 79)	0.027
History of hypertension (year)	46 (30.5)	49 (28.8)	69 (45.7)	0.52
HbA1c (%)	6.9 (6.2, 7.1)	5.43 (5.2, 6.1)	6.72 (6.41, 6.89)	<0.001
FBG (mmol/L)	6.4 (5.1, 7.8)	5.21 (4.71, 5.6)	5.63 (4.78, 5.98)	<0.001
TG (mmol/L)	1.23 (0.91, 1.78)	1.35 (1.1, 1.92)	1.6 (0.95, 1.74)	0.067
TC (mmol/L)	3.62 (3.1, 4.8)	4.43 (3.46, 5)	4.7 (4.16, 5.38)	<0.001
HDL-C (mmol/L)	1.25 (1.04, 1.45)	1.36 (1.06, 1.65)	1.21 (1.01, 1.34)	<0.001
LDL-C (mmol/L)	2.5 (1.87, 3.02)	2.13 (1.76, 2.91)	2.7 (2.42, 3.3)	<0.001
Troponin I (μg/L)	0.008 (0.003, 0.008)	0.006 (0.001, 0.019)	0.032 (0.001, 0.26)	<0.001
Creatinine (μmol/L)	73.2 (56.6, 89.7)	81.2 (66.76, 94.05)	67.13 (56.87, 73.58)	<0.001
Uric acid (μmol/L)	337.03 (337.03 ± 110.72)	320.14 (320.14 ± 111.72)	332.76 (332.76 ± 106.75)	0.018
Albumin (g/L)	38.13 (34.42, 41.31)	37.09 (32.13, 41.36)	39.92 (37.71, 42.55)	<0.001
AST (U/L)	19.1 (15.3, 26.06)	19.36 (15.74, 27.15)	23.23 (16.96, 24.98)	0.462
ALT (U/L)	18.1 (12.11, 29.5)	20.2 (14.32, 28.02)	25.03 (14.15, 26.88)	0.397
GGT (U/L)	22.6 (16.1, 34.5)	19.1 (14.2, 31.25)	34.32 (15.73, 35.16)	0.019
Total bilirubin (μmol/L)	12.2 (9.3, 16.5)	13.71 (10.29, 20)	13.1 (9.8, 15.2)	<0.001
Apolipoprotein A1 (g/L)	1.35 (1.35 ± 0.28)	1.26 (1.26 ± 0.29)	1.29 (1.29 ± 0.27)	<0.001
Apolipoprotein B (g/L)	0.83 (0.66, 1.04)	0.73 (0.56, 0.91)	1.36 (0.9, 1.33)	<0.001
T-wave changes	73 (48.3)	52 (30.6)	71 (46.7)	<0.001
ST-segment change	71(47.02)	60(35.2)	20(13.6)	<0.001

HbA1c, glycated hemoglobin A1c; FBG, fasting blood glucose; TG, triglycerides; TC, total cholesterol; HDL-C, high-density lipoprotein cholesterol; LDL, low-density lipoprotein cholesterol; AST, aspartate aminotransferase; ALT, alanine aminotransferase; GGT, γ-glutamyl transferase.

### 3.2 Selection results of significant indicator combinations

A combination of 8 significant indicators was pinpointed through multifactorial association and significance analysis, demonstrating strong correlation with T2D and CHD: HbA1c, fasting blood glucose, HDL-C, apolipoprotein B, total bilirubin, T-wave change, ST-segment change, and heart rate, detailed in Table 5. These findings form a solid foundation for our diagnostic models.

**Table 5:** Results of multivariate correlation analysis.

Indicators	B	Wald	Significance *P*	OR (95%CI)
HbA1c (%)	−1.468	35.652	<0.001	0.23 (0.142, 0.373)
FBG (mmol/L)	−0.451	13.215	<0.001	0.619 (0.512, 0.735)
HDL-C (mmol/L)	1.969	17.818	<0.001	7.160 (2.871, 17.861)
Apolipoprotein B (g/l)	−4.588	34.331	<0.001	0.010 (0.002, 0.047)
Total bilirubin (μmol/L)	0.060	7.792	0.005	1.061 (1.018, 1.107)
T-wave changes	0.523	10.26	0.017	1.532 (1.251, 3.518)
ST segment changes	0.573	9.428	0.026	1.735 (1.105, 1.529)
Heart rate (bpm)	−0.413	6.312	0.037	0.976 (0.937, 0.999)

### 3.3 Intelligent model typing diagnostic results

Employing 21 sensitive indicators from the univariate analysis and the 8 selected from multifactorial analysis, two diagnostic models were developed: a neural network and a logistic regression model, aimed at classifying T2D with CHD cases.

#### 3.3.1 Neural network diagnostic results

**Figure 2:**
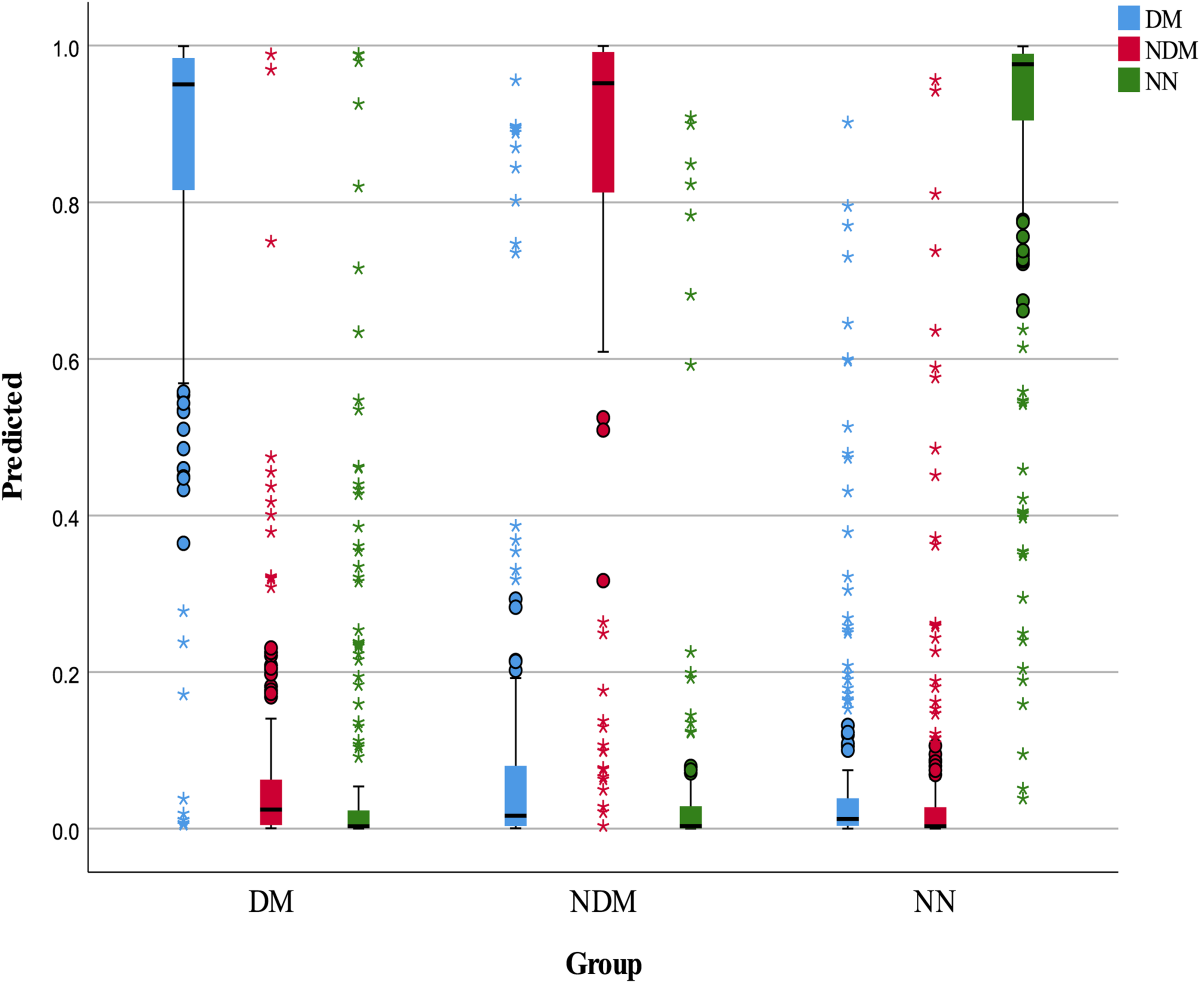
Box plot of neural network classification results. This figure displays the classification performance of the neural network model, represented through a box plot. The color blue indicates predicted T2D-CHD cases, red denotes NDM-CHD cases, and green symbolizes Non-CHD cases.

**Figure 3:**
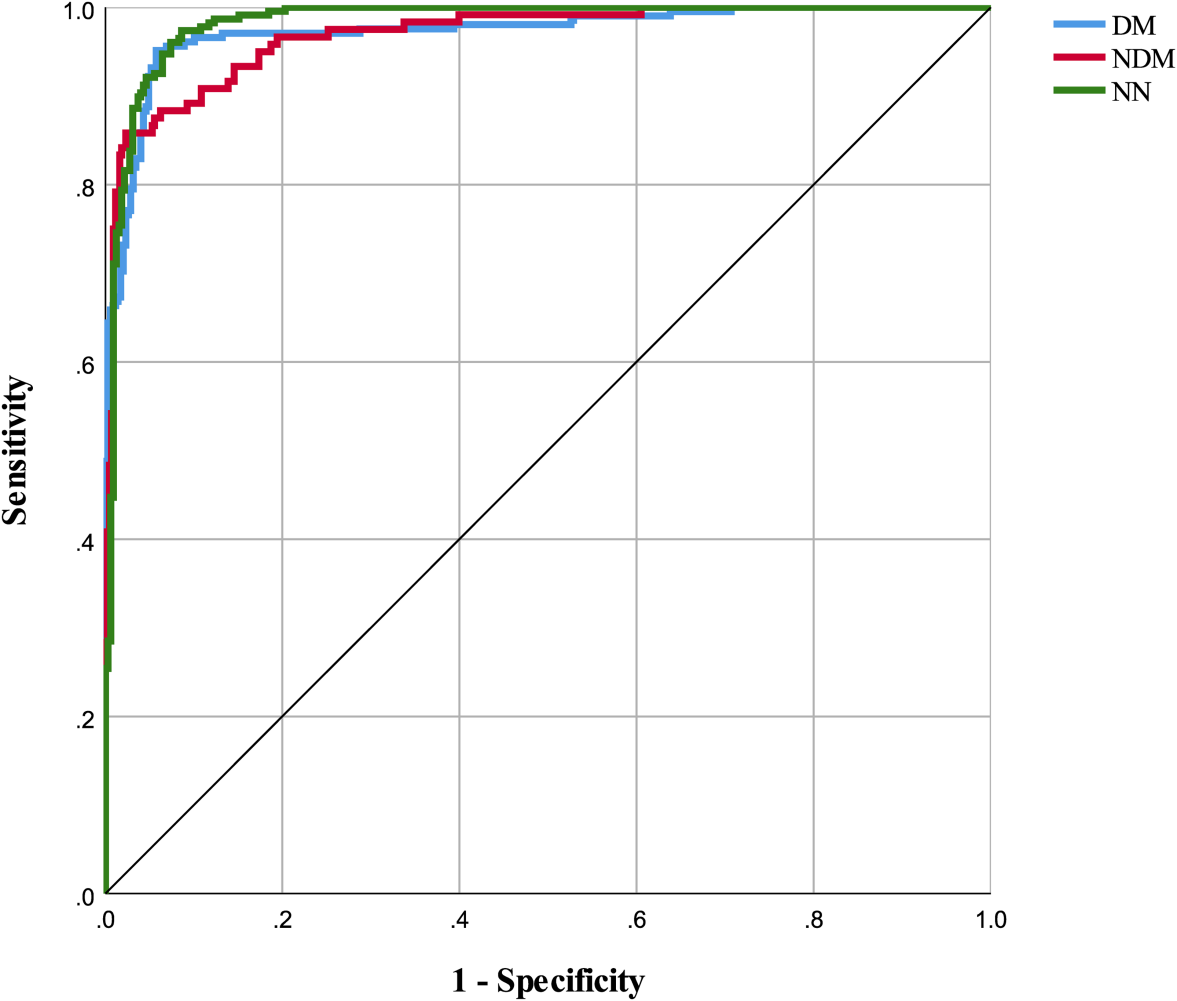
ROC curves for classification indicators in neural network model. This figure displays the ROC (Receiver Operating Characteristic) curves for each classification indicator within the neural network model. The areas under the curves for DM, NDM, and NN are 0.973, 0.968, and 0.982 respectively.

The neural network model was assessed using a test set, with its classification performance depicted in Figure 2. The model distinguished T2D-CHD, CHD-only, and T2D-only groups with notable accuracy, though some outliers were observed. Figure 3 presents ROC curves for each classification indicator, showcasing discriminative capabilities with AUC values of 0.961, 0.964, and 0.977, respectively. Performance metrics, listed in Tables 6, 7, include an accuracy of 90.7%, recall of 90.78%, precision of 90.83%, and an F-1 score of 0.908, highlighting its diagnostic precision.

**Table 6:** Neural network four-fold cross-validation results.

Training set classification results				Test set classification results			
T2D-CHD	CHD-only	T2D-only	Total	T2D-CHD	CHD-only	T2D-only	Total
136	152	144	432	64	68	73	205
139	161	141	441	65	71	68	204
143	149	143	435	62	73	70	205
141	150	142	433	63	70	69	202

**Table 7:** Neural network classification results.

Training set accuracy				Test set accuracy			
Accuracy	Recall	Precision	F-1 score	Accuracy	Recall	Precision	F-1 score
92.02%	92.16%	92.35%	0.923	90.27%	90.78%	90.83%	0.908

#### 3.3.2 Logistic regression diagnostic results

The logistic regression model, applying the 8 chosen indicators, showed proficiency in disease classification, as detailed in Tables 8, 9. It accurately classified the majority of cases across the three categories, achieving an accuracy of 90.13%, recall of 90.1%, precision of 90.22%, and an F-1 score of 0.9016, demonstrating reliable diagnostic capability.

**Table 8:** Logistic regression four-fold cross-validation results.

Training set classification results				Test set classification results			
T2D-CHD	CHD-only	T2D-only	Total	T2D-CHD	CHD-only	T2D-only	Total
136	149	143	428	62	69	71	202
140	150	141	431	64	72	67	203
133	153	139	425	61	70	69	200
138	149	140	427	63	71	70	204

**Table 9:** Logistic classification results.

Training set accuracy				Test set accuracy			
Accuracy	Recall	Precision	F-1 score	Accuracy	Recall	Precision	F-1 score
90.43%	90.81%	91.02%	90.91%	89.49%	90.10%	90.22%	0.9016

### 3.4 External validation of intelligent model typing diagnostic results

External validation assessed the diagnostic models’ generalization capability using data from an independent hospital cohort, which confirmed their effectiveness across T2D-CHD, CHD-only, and T2D-only patient categories, adhering to stringent analytical standards.

#### 3.4.1 Neural network model external validation

The external validation of the neural network model, encapsulated in its classification confusion matrix and summarized in Tables 10, 11, confirmed its exceptional capability to accurately differentiate among the three patient categories. The model achieved an external validation accuracy of 90.57%, with closely aligned recall, precision, and F-1 scores of 90.62%, 90.55%, and 0.9058, respectively, affirming its robust performance and wide applicability.

**Table 10:** Neural network classification confusion matrix.

	T2D-CHD	CHD-only	T2D-only
DM-CHD	67	3	3
CHD-only	3	62	4
T2D-only	5	2	63

**Table 11:** Neural network model external validation results.

Accuracy	Recall	Precision	F-1 score
90.57%	90.62%	90.55%	0.9058

#### 3.4.2 Logistic regression model external validation

Parallelly, the logistic regression model's external validation, meticulously detailed in Tables 12, 13, illustrated its commendable accuracy and consistency in disease state classification. This model's external validation metrics showcased an overall accuracy of 89.15%, complemented by a recall of 89.23%, precision of 89.12%, and an F-1 score of 0.8917, further reinforcing the diagnostic models’ reliability and effectiveness in a broader clinical context.

**Table 12:** Logistic classification confusion matrix.

	T2D-CHD	CHD-only	T2D-only
T2D-CHD	66	2	5
CHD-only	4	59	6
T2D-only	1	5	64

**Table 13:** Logistic model external validation results.

Accuracy	Recall	Precision	F-1 score
89.15%	89.23%	89.12%	0.8917

## 4 Discussion

The necessity of managing cardiovascular risk in patients with Type 2 Diabetes Mellitus (T2D) has become increasingly evident, prompting a shift in the approach to cardiovascular disease (CVD) prevention and management (8). The development of risk assessment tools by authoritative bodies like the American College of Cardiology (ACC) and the American Heart Association (AHA) (9), alongside recommendations from the European Society of Cardiology (ESC) (10), signifies a strategic move towards early detection and primary prevention. These tools and stratifications aim to tailor preventive measures and treatments by evaluating individual risk factors such as age, lifestyle, comorbidities, and disease duration. Yet, the variation in methodologies underscores the complexity of accurately assessing CVD risk in T2D patients, highlighting a critical need for consensus and a more personalized approach to care.

The traditional approach for diagnosing Coronary Heart Disease (CHD) in patients with Type 2 Diabetes Mellitus (T2D) primarily utilizes electrocardiograms (ECG) and coronary angiography. ECGs, as a non-invasive tool, are crucial for detecting heart irregularities and ischemic conditions but may not always accurately capture the onset of CHD or may have ischemic changes masked by other diseases or confounding factors (11). Coronary CT angiography (CTA) represents a non-invasive early screening method, yet it suffers from a higher false-negative rate, making it challenging to widely implement in primary care settings. Meanwhile, Invasive coronary angiography, despite offering detailed artery visualization, is invasive and carries risks, rendering it less suitable for patients in early or asymptomatic stages of CHD (12). Critically, the neuropathic effects of diabetes can conceal the typical pain associated with heart conditions, often leading diabetic patients to delay seeking medical attention and hindering effective detection and diagnosis (13). This underscores an urgent need for advancements in diagnostic methods that are less invasive, highly sensitive, and customized to individual patients, aiming to enhance the early detection and efficient management of CHD in those with T2D.

In response, our study introduces a pioneering sensitivity index screening method and a rapid diagnostic model for T2D-CHD, leveraging the latest advancements in machine learning (ML) and artificial intelligence (AI) to transcend the barriers posed by current diagnostic methodologies. This approach, drawing upon a comprehensive array of medical data, including patient history, comorbidities, laboratory tests, and ECGs, aims to facilitate early detection and risk stratification of CHD among T2D patients. The inspiration for this approach stems from recent breakthroughs in AI/ML, which have demonstrated significant potential in revolutionizing the diagnosis and management of cardiovascular diseases and diabetes.

For instance, the work of Alimova et al., employing ML algorithms to predict diastolic dysfunction in cardiovascular and diabetic patients, highlights the precision and effectiveness these technologies bring to medical diagnostics (14). This is further supported by research from Saeed and Hama, who explored cardiac disease prediction using AI algorithms (15), and from Chinmayi et al. and Barbieri et al., who delved into AI's role in disease risk prediction and the utilization of advanced imaging techniques for enhanced diagnostic accuracy (16, 17). These studies underscore the adaptability and depth of AI/ML in capturing complex cardiovascular and metabolic interrelations, setting a foundation for our methodology.

## 5 Limitations and future directions

While our study presents a promising direction for the use of AI in T2D-CHD diagnostics, we acknowledge limitations such as the reliance on limited training sets and potential selection biases inherent in electronic medical records. Future research could extend our findings through multi-center external dataset validation or prospective cohort studies, further refining the diagnostic models and expanding their applicability.

## 6 Conclusion

Our work contributes to the evolving evidence base supporting the integration of AI and ML in diagnosing complex diseases like T2D-CHD. Our results, in line with recent advancements, advocate for the potential of AI-based diagnostic models to significantly improve disease screening and management, particularly valuable in primary care settings where early detection can dramatically influence patient care and outcomes.

## Data Availability

The raw data supporting the conclusions of this article will be made available by the authors, without undue reservation.
